# Complete sequence of the genome-reduced *Escherichia coli* DGF-298

**DOI:** 10.1128/MRA.00665-23

**Published:** 2023-10-16

**Authors:** Dominick Matteau, Antoine Champie, Frédéric Grenier, Sébastien Rodrigue

**Affiliations:** 1 Département de Biologie, Université de Sherbrooke, Sherbrooke, Québec, Canada; University of Maryland School of Medicine, Baltimore, Maryland, USA

**Keywords:** *Escherichia coli*, DGF-298, whole-genome sequencing, synthetic biology, systems biology, reduced genome

## Abstract

We report the complete genome sequence and annotation of *Escherichia coli* DGF-298, a genome-reduced *E. coli* strain with interesting properties for systems and synthetic biology. DGF-298 has a single circular chromosome of 2,991,126 bp and 2,831 genes, including 2,691 coding sequences, with a mean G + C content of ~51%.

## ANNOUNCEMENT

Genome-reduced organisms such as *Escherichia coli* DGF-298 constitute interesting cell chassis for synthetic biology (1 – 5). DGF-298 was generated from *E. coli* W3110 by deleting non-essential genes, prophages, and other undesirable elements using λ-Red recombination and P1 transduction (2, 3). Approximately 36% of the chromosome was removed while preserving a growth comparable to W3110 (1, 2, 5). We proceeded to the whole-genome sequencing of *E. coli* DGF-298 using Illumina sequencing and Oxford Nanopore Technology (ONT).

DGF-298 (DGF-298W100::rev234::SC) was acquired from the KHK Collection of the SHIGEN National Institute of Genetics. DGF-298 genomic DNA (gDNA) was isolated from an overnight culture grown in LB medium at 37°C using the Quick-gDNA MiniPrep kit (Zymo Research) according to the manufacturer’s specifications. To prepare an ONT sequencing library, 1.5 µg of gDNA was sheared at ~20 kbp using a Covaris g-TUBE by centrifuging twice at 1,650 × *g* for 1 min. No further size selection was performed. Sequencing adaptors were ligated using the R9 SQK-NSK007 ONT Ligation Sequencing Kit, and sequencing was performed on a MinION Mk1B device equipped with an R9 flow cell. Live base-calling was performed using MinKNOW software v1.1.21. A total of 1,614 reads passed MinKNOW default quality filter, corresponding to 14,978,836 bp with an *N*_50_ of 13,742 bp. For Illumina sequencing, DNA was sheared and prepared using the QIAseq FX DNA Library Kit (QIAGEN) with 100 ng of the same gDNA preparation according to the manufacturer’s specifications, with the following modifications: (i) 2 min fragmentation time, (ii) provided adaptors and primers replaced by custom oligonucleotides, (iii) library amplification using the VeraSeq 2.0 DNA polymerase (Enzymatics), and (iv) final library purification and size selection using 0.7× AMPure XP beads (Agencourt). Library quality was assessed on a 2100 Bioanalyzer instrument (Agilent). Paired-end Illumina sequencing (2 × 125 bp) was performed on an Illumina HiSeq 2000 system at the McGill University and Génome Québec Innovation Centre (Montréal, Canada). 1,308,045 paired-end reads were obtained, corresponding to a genome coverage of ~100×. Illumina read quality was evaluated with FastQC v0.11.9, revealing an average Phred score of 35 for the forward and reverse reads. DGF-298 genome was *de novo* assembled using quality-filtered ONT reads and Newbler 2.6 RunAssembly, generating a single scaffold of 2,966,096 bp comprising seven contigs. Gaps were filled and circular conformation was confirmed by performing local mapping of ONT and Illumina reads using Newbler 2.6 RunMapping. Adapters were trimmed using Newbler 2.6 vt option. Genome assembly was error-corrected by alignment of the Illumina reads using BWA sampe v0.7.15 (6). DGF-298 chromosome was manually rotated to match *E. coli* W3110 (AP009048.1) start and end coordinates. Genome annotations were transferred from *E. coli* W3110 to the corrected sequence with RATT 1.0 (7), and mutations were identified using MUMmer v3.23 (8) (Table 1). Annotations were manually curated according to the detected mutations and deleted regions present in DGF-298 (2, 3).

**TABLE 1:** Complete genomic changes between *E. coli* DGF-298 and W3110

Mutation type^*a*^	Start position DGF-298^*b*^	End position DGF-298^*b*^	Length (bp)	Start position W3110^*c*^	End position W3110^*c*^	Length (bp)	Nucleotide change^*d*^	Deleted gene(s)^*e*^	Partially affected gene(s)	Protein mutation type(s)	Protein mutation(s)	Notes^*f*^
Deletion	15445	15445	0	15445	16903	1,459	n/a	*insL; hokC*	*mokC*	Deletion	p.Met20_Glu69del	*mokC* is now annotated as pseudo
Deletion	18187	18187	0	19646	20508	863	n/a	*insB; insA*	-	-	-	-
Deletion	63535	63535	0	65857	78797	12,941	n/a	*araD; araA; araB; araC; yabI; thiQ; thiP; tbpA; sgrR; sgrS; setA*	-	-	-	-
Insertion	63540	63542	3	78802	78802	0	n/a	-	Intergenic	-	-	-
Substitution	140203	140203	1	155463	155463	1	A > T	-	*ecpD*	Extension	p.*247Glyext*11	Mutation in stop codon, *ecpD* is now annotated as pseudo
Deletion	152141	152141	0	167401	173315	5,915	n/a	*fhuA; fhuC; fhuD*	*fhuB*	Deletion	p.Met1_Gly618del	*fhuB* is now annotated as pseudo
Deletion	223125	223125	0	244301	253746	9,446	n/a	*yafJ; yafK; yafQ; dinJ; yafL; yafM; fhiA; mbhA; dinB; yafN; yafO; yafP; ykfJ*	*prfH*	Deletion	p.Met1_Ser15del	*prfH* is now annotated as pseudo
Substitution	223627	223627	1	254249	254249	1	A > T	-	Intergenic	-	-	-
Deletion	231678	231678	0	262300	387867	125,568	n/a	*ykfI; yafW; ykfH; ykfG; yafX; ykfF; ykfB; yafY; yafZ; ykfA; perR; insN; insI; insO; ykfC; insH; mmuP; mmuM; afuC; afuB; insB; insA; ykgN; yagB; yagA; yagE; yagF; yagG; yagH; yagI; argF; insB; insA; yagJ; yagK; yagL; yagM; yagN; intF; yagP; yagQ; yagR; yagS; yagT; yagU; ykgJ; yagV; yagW; yagX; yagY; yagZ; ykgK; ykgL; ykgM; eaeH; insE; insF; ykgA; ykgB; ykgI; ykgC; ykgD; ykgE; ykgF; ykgG; ykgH; betA; betB; betI; betT; yahA; yahB; yahC; yahD; yahE; yahF; yahG; yahH; yahI; yahJ; yahK; yahL; yahM; yahN; yahO; prpR; prpB; prpC; prpD; prpE; codB; codA; cynR; cynT; cynS; cynX; lacA; lacY; lacZ; lacI; mhpR; mhpA; mhpB; mhpC; mhpD; mhpF; mhpE; mhpT; yaiL; frmB; frmA; frmR; yaiO; yaiX; insC; insD; yaiF; yaiP; yaiS; tauA; tauB; tauC; tauD*	-	-	-	-
Substitution	232635	232635	1	388825	388825	1	C > T	-	*hemB*	Missense	p.Asp43Asn	-
Substitution	232901	232901	1	389091	389091	1	T > C	-	Intergenic	-	-	-
Deletion	233285	233285	0	389475	404039	14,565	n/a	*yaiT; insF; insE; yaiU; yaiV; ampH; sbmA; yaiW; yaiY; yaiZ; ddlA; yaiB; phoA; psiF; yaiC*	-	-	-	-
Substitution	327386	327386	1	498141	498141	1	T > C	-	*hemH*	Missense	p.Phe288Ser	-
Deletion	347612	347612	0	518367	533048	14,682	n/a	*tesA; ybbA; ybbP; rhsD; ybbC; ylbH; ybbD; ylbG; ybbB; ybbS; allA; allR*	*ybbO*	Deletion	p.Met1_His3del	*ybbO* is now annotated as pseudo
Substitution	349124	349124	1	534561	534561	1	T > C	-	*gcl*	Silent	p.Ala474Ala	-
Substitution	349960	349960	1	535397	535397	1	T > C	-	*hyi*	Missense	p.Leu155Pro	-
Substitution	350279	350279	1	535716	535716	1	A > G	-	Intergenic	-	-	-
Substitution	350336	350336	1	535773	535773	1	T > C	-	Intergenic	-	-	-
Deletion	350404	350404	0	535841	550551	14,711	n/a	*ybbV; ybbW; allB; ybbY; glxK; ylbA; allC; allD; fdrA; ylbE; ylbF; ybcF*	*glxR*	Deletion	p.Met11_Ala292del	*glxR* is now annotated as pseudo
Substitution	356710	356710	1	556858	556858	1	A > T	-	*folD*	Missense	p.Leu36Gln	-
Deletion	364129	364129	0	564277	608454	44,178	n/a	*ybcC; ybcD; insE; insF; renD; emrE; ybcK; ybcL; ybcM; ybcN; ninE; ybcO; rusA; ylcG; ybcQ; insH; nmpC; essD; ybcS; rzpD; rzoD; borD; ybcV; ybcW; nohB; tfaD; ybcY; ylcE; appY; ompT; envY; ybcH; nfrA; nfrB; cusS; cusR; cusC; cusF; cusB; cusA; pheP; ybdG; nfnB; ybdF; ybdJ; ybdK; hokE; insL*	*intD*	Deletion	p.Met1_Asp310del	*intD* is now annotated as pseudo
Substitution	364131	364131	1	608457	608457	1	G > A	-	Intergenic	-	-	-
Deletion	396343	396343	0	640669	659192	18,524	n/a	*uspG; ybdR; rnk; rna; citT; citG; citX; citF; citE; citD; citC; citA; citB; 'dcuC; insH; dcuC'; crcA; cspE; crcB; ybeH*	*ybeM*	Deletion	p.Met1_Gln172del	*ybeM* is now annotated as pseudo
Substitution	412090	412090	1	674940	674940	1	G > A	-	*leuS*	Missense	p.Ala89Val	-
Substitution	412492	412492	1	675342	675342	1	T > C	-	Intergenic	-	-	-
Substitution	412564	412564	1	675414	675414	1	C > A	-	Intergenic	-	-	-
Deletion	412590	412590	0	675440	689710	14,271	n/a	*ybeL; ybeQ; ybeR; djlB; ybeT; ybeU; djlC; hscC; rihA; gltL; gltK; gltJ; gltI; insH*	-	-	-	-
Deletion	452036	452036	0	729157	739929	10,773	n/a	*kdpF; ybfA; rhsC; ybfB; ybfO; ybfC; ybfQ; ybfL; ybfD; ybgA*	*phr*	Unknown	p.Met1?	Mutation in translation start codon, *phr* is now annotated as pseudo
Deletion	459251	459251	0	747145	753217	6,073	n/a	*abrB; ybgO; ybgP; ybgQ; ybgD*	*nei*	Missense and extension	p.His263Gln and p.*264Thrext*9	Mutation in stop codon, *nei* is now annotated as pseudo
Deletion	538922	538922	0	832889	848426	15,538	n/a	*ybiA; dinG; ybiB; ybiC; ybiJ; ybiI; ybiX; fiu; ybiM; ybiN; ybiO; glnQ; glnP; glnH*	-	-	-	-
Deletion	558470	558470	0	867975	883810	15,836	n/a	*yliA; yliB; yliC; yliD; yliE; yliF; yliG; yliH; yliI; yliJ; dacC; deoR; ybjG*	-	-	-	-
Deletion	565994	565994	0	891335	909716	18,382	n/a	*ybjC; nfsA; rimK; ybjN; potF; potG; potH; potI; ybjO; rumB; artJ; artM; artQ; artI; artP; ybjP; ybjQ; ybjR; ybjS; ybjT; ltaE*	-	-	-	-
Substitution	705295	705295	1	1049018	1049018	1	G > A	-	*ymcB*	Silent	p.Gly32Gly	-
Deletion	705504	705504	0	1049227	1097311	48,085	n/a	*ymcD; insA; insB; cspH; cspG; ymcE; gnsA; yccM; torS; torT; torR; torC; torA; torD; cbpM; cbpA; yccE; agp; yccJ; wrbA; ymdF; ycdG; ycdH; ycdI; rarA; ycdK; ycdL; ycdM; ycdC; putA; putP; ycdN; ycdO; ycdB; phoH; ycdP; ycdQ; ycdR; ycdS; ycdT; insF; insE; ymdE; ycdU*	*ymcC*	Deletion	p.Met1_Lys176del	*ymcC* is now annotated as pseudo
Deletion	706320	706320	0	1098128	1117068	18,941	n/a	*ycdW; ycdX; ycdY; ycdZ; csgG; csgF; csgE; csgD; csgB; csgA; csgC; insD; insC; ymdA; ymdB; ymdC; mdoC; mdoG; mdoH; yceK; msyB; mdtG*	-	-	-	-
Substitution	706680	706680	1	1117429	1117429	1	T > C	-	*lpxL*	Missense	p.Lys244Arg	-
Substitution	720216	720216	1	1130965	1130965	1	C > T	-	Intergenic	-	-	-
Deletion	720243	720243	0	1130992	1142564	11,573	n/a	*flgN; flgM; flgA; flgB; flgC; flgD; flgE; flgF; flgG; flgH; flgI; flgJ; flgK; flgL*	-	-	-	-
Substitution	776116	776116	1	1198438	1198438	1	A > G	-	Intergenic	-	-	-
Deletion	776122	776122	0	1198444	1225484	27,041	n/a	*ymfD; ymfE; lit; intE; ymfG; ymfH; ymfI; ymfJ; ymfK; ymfT; ymfL; ymfM; ymfN; ymfR; ymfO; ymfP; ymfQ; ycfK; ymfS; tfaE; stfE; pin; mcrA; icdC; elbA; ycgX; ycgE; ycgF; ycgZ; ymgA; ymgB; ymgC; ycgG; ymgF; ycgH; ymgD; ymgG; ymgH; ycgI*	-	-	-	-
Deletion	821463	821463	0	1270827	1271361	535	n/a	*rdlA; IdrB*	*ldrA*	Unknown	p.Met1?	Mutation in translation start codon, *ldrA* is now annotated as pseudo
Deletion	846105	846105	0	1296003	1308864	12,862	n/a	*ychG; adhE; ychE; insC; insD; oppA; oppB; oppC; oppD; oppF; yciU*	-	-	-	-
Deletion	896757	896757	0	1359517	1368530	9,014	n/a	*puuP; puuA; puuD; puuR; puuC; puuB; puuE*	-	-	-	-
Deletion	924831	924831	0	1396605	1520045	123,441	n/a	*ynaI; insH; ynaJ; uspE; fnr; ogt; abgT; abgB; abgA; abgR; isrA; ydaL; ydaM; ydaN; dbpA; ydaO; intR; ydaQ; ydaC; lar; recT; recE; racC; ydaE; kil; sieB; ydaF; ydaG; racR; ydaS; ydaT; ydaU; ydaV; ydaW; rzpR; rzoR; trkG; ynaK; ydaY; ynaA; lomR'; insH; 'lomR; stfR; tfaR; pinR; ynaE; uspF; ompN; micC; ydbK; ydbJ; hslJ; ldhA; ydbH; ynbE; ydbL; feaR; feaB; tynA; maoC; paaA; paaB; paaC; paaD; paaE; paaF; paaG; paaH; paaI; paaJ; paaK; paaX; paaY; ydbA'; insD; insC; insI; 'ydbA; ydbC; ydbD; ynbA; ynbB; ynbC; ynbD; azoR; hrpA; ydcF; aldA; gapC; cybB; ydcA; hokB; mokB; sokB; trg; ydcI; ydcJ; mdoD; ydcH; rimL; ydcK; tehA; tehB; ydcL; yncK; ydcM; ydcO; ydcN; ydcP; yncJ; yncN; ydcQ; ydcR; ydcS; ydcT; ydcU; ydcV; ydcW; ydcX; ydcY; ydcZ*	*yncA*	Extension	p.*173Serext*24	Mutation in stop codon, *yncA* is now annotated as pseudo
Deletion	927172	927172	0	1522387	1543782	21,396	n/a	*yncD; yncE; ansP; yncG; yncH; rhsE; ydcD; yncI; yncM; ydcC; ydcE; yddH; nhoA; yddE; narV; narW; narY*	*yncC; narZ*	Deletion; Deletion	p.Phe138_Arg221del; p.Val175_Lys1246del	*yncC* and *narZ* are now annotated as pseudos
Substitution	965844	965844	1	1582455	1582455	1	T > A	-	*ydeM*	Missense	p.Asp17Val	-
Deletion	965946	965946	0	1582557	1592251	9,695	n/a	*ydeN; ydeO; ydeP; ydeQ; ydeR; ydeS; ydeT; yneL*	-	-	-	-
Deletion	966262	966262	0	1592568	1599800	7,233	n/a	*hipA; hipB; ydeU; ydeK*	-	-	-	-
Deletion	974263	974263	0	1607803	1607803	1	n/a	Intergenic	Intergenic	-	-	-
Deletion	974283	974283	0	1607823	1624231	16,409	n/a	*IsrF; lsrG; tam; yneE; uxaB; yneF; yneG; yneH; yneI; yneJ; yneK; ydeA; marC; marR; marA; marB; eamA; ydeE*	-	-	-	-
Substitution	974990	974990	1	1624939	1624939	1	T > A	-	*ydeH*	n/a	n/a	Irrelevant mutation, presence of an upstream frameshift in CDS
Deletion	975258	975258	0	1625208	1625208	1	n/a	-	*ydeH*	Frameshift	p.Thr120Tyrfs*6	*ydeH* is now annotated as pseudo
Substitution	980096	980096	1	1630046	1630046	1	T > C	-	Intergenic	-	-	-
Deletion	980104	980104	0	1630054	1640180	10,127	n/a	*ydfH; ydfZ; ydfI; ydfJ; ydfK; pinQ; tfaQ; stfQ; nohA; ynfO; ydfO; gnsB; ynfN*	*cspI*	Deletion	p.Val68_Leu70del	*cspI* is now annotated as pseudo
Substitution	980208	980208	1	1640285	1640285	1	G > A	-	*cspI*	Missense	p.His33Tyr	-
Insertion	981683	981683	1	1641759	1641759	0	n/a	-	*ydfQ*	Frameshift	p.Ile5Aspfs*24	*ydfQ* is now annotated as pseudo
Deletion	981695	981695	0	1641771	1649361	7,591	n/a	*ydfR; essQ; cspB; cspF; ydfT; ydfU; rem; hokD; relE; relB; ydfV; flxA; ydfW; ydfX*	*ydfQ; dicC*	Unknown; deletion	p.Met1?; p.Arg69_Ser76del	Mutation in *ydfQ* start codon; *ydfQ* and *dicC* are now annotated as pseudos
Deletion	983841	983841	0	1651508	1657863	6,356	n/a	*ydfD; ydfE; insD; intQ; ynfP; rspB; rspA; ynfA; ynfB*	*dicB*	Missense and extension	p.Gln62His and p.*63Leuext*5	Mutation in stop codon, *dicB* is now annotated as pseudo
Substitution	1036505	1036505	1	1710528	1710528	1	A > G	-	*rsxC*	Silent	p.Ala632Ala	-
Substitution	1179532	1179532	1	1853555	1853555	1	C > T	-	*ansA*	Nonsense	p.Gln328*	New upstream stop codon, *ansA* is now annotated as pseudo
Deletion	1179557	1179557	0	1853580	1864142	10,563	n/a	*pncA; ydjE; ydjF; ydjG; ydjH; ydjI; ydjJ; ydjK; ydjL; yeaC; yeaA*	*ansA*	n/a	n/a	Irrelevant mutation, presence of a new upstream stop codon in CDS
Deletion	1190702	1190702	0	1875288	1888499	13,212	n/a	*yeaK; yoaI; yeaL; yeaM; yeaN; yeaO; yoaF; yeaP; yeaQ; yoaG; yeaR; yeaS; yeaT; yeaU; yeaV; yeaW; yeaX*	-	-	-	-
Substitution	1190704	1190704	1	1888502	1888502	1	A > G	-	*yeaX*	n/a	n/a	Irrelevant mutation, presence of another mutation (del) that causes gene deletion
Substitution	1191074	1191074	1	1888872	1888872	1	C > A	-	*rnd*	n/a	n/a	Irrelevant mutation, presence of a new upstream stop codon in CDS
Substitution	1191560	1191560	1	1889358	1889358	1	C > T	-	*rnd*	Nonsense	p.Trp116*	New upstream stop codon, *rnd* is now annotated as pseudo
Substitution	1250792	1250792	1	1948590	1948590	1	C > A	-	*ruvC*	Silent	p.Leu167Leu	-
Deletion	1265866	1265866	0	1963664	1992215	28,552	n/a	*yecT; flhE; flhA; flhB; cheZ; cheY; cheB; cheR; tap; tar; cheW; cheA; motB; motA; flhC; flhD; insH; yecG; otsA; otsB; araH; araG; araF; yecI; yecJ; isrB; yecR; ftn; yecH*	*tyrP*	Deletion	p.Met1_Gly133del	*tyrP* is now annotated as pseudo
Deletion	1269451	1269451	0	1995801	2027348	31,548	n/a	*uvrY; yecF; sdiA; yecC; yecS; yedO; fliY; fliZ; fliA; fliC; fliD; fliS; fliT; amyA; yedD; yedE; yedF; yedK; yedL; yedN; yedM; intG; fliE; fliF; fliG; fliH; fliI; fliJ; fliK; fliL; fliM; fliN; fliO; fliP; fliQ; fliR; rcsA; dsrB; yodD*	*uvrC*	Deletion	p.Met1_Arg348del	*uvrC* is now annotated as pseudo
Deletion	1274690	1274690	0	2032588	2043515	10,928	n/a	*vsr; dcm; yedJ; yedR; yedS; hchA; yedV; yedW; yedX; yedY; yedZ*	*yedA; yodA*	Missenses and extension; Deletion	p.Ser305Arg, p.Glu306Phe, and p.*307Valext*9; p.Met1_Ala2del	*yedA* and *yodA* are now annotated as pseudos
Substitution	1274778	1274778	1	2043604	2043604	1	A > G	-	*yodA*	Silent	p.Lys31Lys	-
Deletion	1299463	1299463	0	2068289	2081066	12,778	n/a	*insH; yoeA; insD; insC; yoeE; yeeP; isrC; flu; yeeR; yeeS; yeeT; yeeU; yeeV; yeeW; yoeF*	-	-	-	-
Substitution	1299487	1299487	1	2081091	2081091	1	A > G	-	Intergenic	-	-	-
Deletion	1309749	1309749	0	2091353	2091607	255	n/a	*yoeB*	*yefM*	Deletion	p.Ile82_Glu83del	Deletion of stop codon, *yefM* is now annotated as pseudo
Substitution	1309751	1309751	1	2091610	2091610	1	T > C	-	*yefM*	Missense	p.Ile81Val	-
Substitution	1309899	1309899	1	2091759	2091759	1	C > G	-	*yefM*	Silent	p.Arg31Arg	-
Substitution	1314255	1314255	1	2096114	2096114	1	C > T	-	*hisB*	Silent	p.His170His	-
Deletion	1321673	1321673	0	2103532	2115203	11,672	n/a	*'wbbL; insH; wbbL'; wbbK; wbbJ; wbbI; wbbH; glf; rfbX; rfbC; rfbA; rfbD; rfbB*	-	-	-	-
Substitution	1321783	1321783	1	2115314	2115314	1	A > G	-	Intergenic	-	-	-
Substitution	1337480	1337480	1	2131011	2131011	1	G > A	-	*wcaF*	Silent	p.Ser5Ser	-
Deletion	1351872	1351872	0	2145403	2186994	41,592	n/a	*yegE; alkA; yegD; yegI; yegJ; yegK; yegL; ryeC; ryeD; mdtA; mdtB; mdtC; mdtD; baeS; baeR; yegP; yegQ; ryeE; ogrK; yegZ; yegR; yegS; 'gatR; insE; insF; gatR'; gatD; gatC; gatB; 'gatA; insH; gatA'; gatZ; gatY; fbaB; yegT; yegU; yegV; yegW; yegX*	-	-	-	-
Deletion	1364685	1364685	0	2199808	2228989	29,182	n/a	*molR'; 'molR; yehI; yehK; yehL; yehM; yehP; yehQ; yehR; yehS; yehT; yehU; mlrA; yohO; yehW; yehX; yehY; yehZ; bglX; dld; pbpG; yohC*	-	-	-	-
Substitution	1390615	1390615	1	2254920	2254920	1	G > A	-	*nfo*	Silent	p.Gln249Gln	-
Deletion	1390730	1390730	0	2255035	2262631	7,597	n/a	*yeiI; yeiJ; rihB; yeiL; yeiM; yeiN; yeiC*	-	-	-	-
Deletion	1417822	1417822	0	2289724	2293603	3,880	n/a	*yejO; insH*	-	-	-	-
Deletion	1445012	1445012	0	2320795	2322130	1,336	n/a	*insD; insC*	*rcsC*	CDS fusion	Fusion of rcsC split coding regions (RcsC:p.Met1_Asp791; RcsC:p.Met1_*125)	Deletion of IS2 insertion element and restoration of *rcsC* found in MG1655 (933 amino acids)
Deletion	1484456	1484456	0	2361574	2383930	22,357	n/a	*yfaD; ypaA; yfaU; yfaV; yfaW; yfaX; yfaY; yfaZ; yfaO; ais; yfbE; yfbF; yfbG; yfbH; arnT; yfbW; yfbJ; pmrD; menE; menC; menB; yfbB; menD*	-	-	-	-
Substitution	1484894	1484894	1	2384368	2384368	1	C > T	-	*menF*	Missense	p.Val316Met	-
Deletion	1485927	1485927	0	2385401	2394610	9,210	n/a	*elaB; elaA; elaC; elaD; yfbK; yfbL; yfbM; yfbN; yfbO; yfbP*	-	-	-	-
Substitution	1486279	1486279	1	2394963	2394963	1	C > T	-	*nuoN*	Missense	p.Ala345Thr	-
Substitution	1502353	1502353	1	2411037	2411037	1	A > G	-	*IrhA*	Missense	p.Leu92Ser	-
Deletion	1502775	1502775	0	2411459	2412234	776	n/a	*insB; insA*	-	-	-	-
Substitution	1502847	1502847	1	2412307	2412307	1	T > A	-	Intergenic	-	-	-
Substitution	1513018	1513018	1	2422478	2422478	1	C > T	-	Intergenic	-	-	-
Substitution	1513033	1513033	1	2422493	2422493	1	A > C	-	Intergenic	-	-	-
Deletion	1513044	1513044	0	2422504	2433237	10,734	n/a	*yfcC; yfcD; yfcE; yfcF; yfcG; folX; yfcH; yfcI; hisP; hisM; hisQ; hisJ; argT*	-	-	-	-
Deletion	1551791	1551791	0	2471986	2471986	1	n/a	Intergenic	Intergenic	-	-	-
Deletion	1551796	1551796	0	2471991	2481621	9,631	n/a	*intS; yfdG; yfdH; yfdI; tfaS; yfdK; yfdL; yfdM; yfdN; yfdO; yfdP; yfdQ; yfdR; yfdS; yfdT*	ypdJ	Deletion	p.Met1_Ile28del	*ypdJ* is now annotated as pseudo
Substitution	1552249	1552249	1	2482075	2482075	1	A > G	-	Intergenic	-	-	-
Substitution	1557415	1557415	1	2487241	2487241	1	G > A	-	*emrY*	Nonsense	p.Gln128*	New upstream stop codon, *emrY* is now annotated as pseudo
Deletion	1564862	1564862	0	2494688	2513688	19,001	n/a	*yfdV; oxc; frc; yfdX; ypdI; yfdY; ddg; yfdZ; ypdA; ypdB; ypdC; ypdD; ypdE; ypdF; ypdG; ypdH*	-	-	-	-
Substitution	1564887	1564887	1	2513714	2513714	1	T > C	-	Intergenic	-	-	-
Substitution	1564912	1564912	1	2513739	2513739	1	A > G	-	Intergenic	-	-	-
Deletion	1570891	1570891	0	2519718	2523278	3,561	n/a	*insL; yfeA*	-	-	-	-
Deletion	1647470	1647470	0	2599858	2613439	13,582	n/a	*hyfA; hyfB; hyfC; hyfD; hyfE; hyfF; hyfG; hyfH; hyfI; hyfJ; hyfR; focB*	-	-	-	-
Deletion	1788845	1788845	0	2754815	2788618	33,804	n/a	*intA; yfjH; alpA; yfjI; yfjJ; yfjK; yfjL; yfjM; yfjN; yfjO; yfjP; yfjQ; yfjR; ypjK; yfjS; yfjT; yfjU; ypjL; yfjV; ypjM; yfjW; yfjX; yfjY; ypjJ; yfjZ; ypjF; ypjA; pinH; ypjB; ypjC; ileY; ygaQ; ygaR; yqaC; yqaD; ygaT*	-	-	-	-
Substitution	1789035	1789035	1	2788809	2788809	1	C > T	-	*ygaF*	Missense	p.Pro58Ser	-
Substitution	1789192	1789192	1	2788966	2788966	1	C > T	-	*ygaF*	Missense	p.Ala110Val	-
Deletion	1794538	1794538	0	2794312	2800028	5,717	n/a	*csiR; ygaU; yqaE; ygaV; ygaP; stpA; ygaW; ygaC; ygaM; nrdH; nrdI*	*nrdE*	Deletion	p.Met1_Leu9del	*nrdE* is now annotated as pseudo
Deletion	1818961	1818961	0	2824452	2829398	4,947	n/a	*srlA; srlE; srlB; srlD; gutM; srlR*	*gutQ*	Deletion	p.Met1_Ile310del	*gutQ* is now annotated as pseudo
Substitution	1826256	1826256	1	2836694	2836694	1	A > G	-	*hydN*	Missense	p.Val23Ala	-
Deletion	1826472	1826472	0	2836910	2855462	18,553	n/a	*ascG; ascF; ascB; hycI; hycH; hycG; hycF; hycE; hycD; hycC; hycB; hycA; hypA; hypB; hypC; hypD; hypE; fhlA; ygbA*	-	-	-	-
Substitution	1837119	1837119	1	2866110	2866110	1	T > A	-	Intergenic	-	-	-
Deletion	1847638	1847638	0	2876630	2876812	183	n/a	Intergenic	Intergenic	-	-	Highly repeated region
Replacement	1892020	1895203	3,184	2921194	2943638	22,445	n/a	*yqcA; yqcB; yqcC; csrB; syd; yqcD; ygdH; sdaC; sdaB; exo; fucO; fucA; fucP; fucI; fucK; fucU; fucR; ygdE; ygdD; gcvA; gcvB; ygdI; csdA; ygdK*	-	Replacement by a sacB and cmR cassette	-	-
Substitution	1935947	1935947	1	2984382	2984382	1	A > G	-	Intergenic	-	-	-
Substitution	1936037	1936037	1	2984472	2984472	1	T > C	-	Intergenic	-	-	-
Deletion	1936063	1936063	0	2984498	3032266	47,769	n/a	*yqeG; yqeH; yqeI; yqeJ; yqeK; ygeF; ygeG; ygeH; ygeI; pbl; ygeK; ygeL; ygeM; ygeN; ygeO; insD; insC; ygeP; ygeQ; glyU; ygeR; xdhA; xdhB; xdhC; ygeV; ygeW; ygeX; ygeY; hyuA; yqeA; yqeB; yqeC; ygfJ; ygfK; ssnA; ygfM; xdhD; ygfO; guaD; ygfQ; ygfS; ygfT; ygfU; idi*	-	-	-	-
Deletion	1961819	1961819	0	3058023	3068464	10,442	n/a	*yqfE; argP; yliK; argK; ygfG; ygfH; ygfI; yggE; argO; mscS*	-	-	-	-
Deletion	1969578	1969578	0	3076224	3078055	1,832	n/a	*cmtB*	*cmtA*	Deletion	p.Met1_Arg431del	*cmtA* is now annotated as pseudo
Substitution	1993418	1993418	1	3101896	3101896	1	G > A	-	*mutY*	Silent	p.Leu76Leu	-
Deletion	2000769	2000769	0	3109247	3133480	24,234	n/a	*yghD; yghE; yghF; yghG; pppA; yghJ; yghK; glcB; glcG; glcF; glcE; glcD; glcC; yghO; insH; yghQ; yghR; yghS; yghT*	-	-	-	-
Deletion	2032058	2032058	0	3164770	3172081	7,312	n/a	*ygiS; ygiT; ygiU; ygiV; ygiW; qseB; qseC; ygiZ; mdaB; ygiN*	-	-	-	-
Substitution	2032063	2032063	1	3172087	3172087	1	T > A	-	*ygiN*	n/a	n/a	Irrelevant mutation, presence of another mutation (del) that causes gene deletion
Deletion	2043763	2043763	0	3183787	3188521	4,735	n/a	*ygiL; insC; insD; yqiG*	-	-	-	-
Substitution	2043897	2043897	1	3188656	3188656	1	T > C	-	*yqiH*	Silent	p.Asp40Asp	-
Substitution	2044135	2044135	1	3188894	3188894	1	A > T	-	*yqiH*	Nonsense	p.Lys120*	New upstream stop codon, *yqiH* is now annotated as pseudo
Substitution	2044622	2044622	1	3189381	3189381	1	A > G	-	*yqiI*	Missense	p.Asn32Asp	-
Substitution	2080125	2080125	1	3224884	3224884	1	T > C	-	Intergenic	-	-	-
Deletion	2080131	2080131	0	3224890	3240400	15,511	n/a	*ygjI; ygjJ; ygjK; fadH; ygjM; ygjN; ygjO; ygjP; ygjQ; ygjR; sraF; alx; sstT; ygjV*	-	-	-	-
Substitution	2090850	2090850	1	3251120	3251120	1	T > C	-	*yhaH*	Missense	p.Ile54Thr	-
Deletion	2098089	2098089	0	3258359	3269672	11,314	n/a	*tdcF; tdcE; insH; tdcD; tdcC; tdcB; tdcA; tdcR; yhaB; yhaC*	-	-	-	-
Insertion	2136951	2136956	6	3308534	3308534	0	n/a	-	*nlpI*	Insertion	p.Arg82_Asn83insAsnAsp	-
Deletion	2189503	2189503	0	3361081	3370891	9,811	n/a	*yhcA; yhcD; yhcE'; insH; 'yhcE; yhcF; yhcG; yhcH; nanK; nanE*	*gltF*	Deletion	p.Ser17_Leu254del	*gltF* is now annotated as pseudo
Substitution	2229132	2229132	1	3410521	3410521	1	C > G	-	*dusB*	Silent	p.Val129Val	-
Deletion	2230110	2230110	0	3411500	3421220	9,721	n/a	*yhdJ; yhdU; envR; acrE; acrF; yhdV; yhdW; yhdX*	*yhdY*	Deletion	p.Met1_Ser14del	*yhdY* is now annotated as pseudo
Substitution	2230111	2230111	1	3421221	3421221	1	C > A	-	*yhdY*	n/a	n/a	Irrelevant mutation, presence of another mutation (del) that causes lost of translation start codon and partial gene deletion
Substitution	2230119	2230119	1	3421229	3421229	1	C > T	-	*yhdY*	n/a	n/a	Irrelevant mutation, presence of another mutation (del) that causes lost of translation start codon and partial gene deletion
Deletion	2295318	2295318	0	3486428	3496908	10,481	n/a	*yijP; yijO; frwD; pflC; pflD; frwB; frwC*	*ptsA*	Deletion	p.Met1_Ala817del	*ptsA* is now annotated as pseudo
Substitution	2295319	2295319	1	3496910	3496910	1	C > A	-	*ptsA*	n/a	n/a	Irrelevant mutation, presence of another mutation (del) that causes lost of translation start codon and near complete gene deletion
Deletion	2295581	2295581	0	3497173	3497173	1	n/a	-	*fsaB*	Frameshift	p.Met68_Trpfs*17	*fsaB* is now annotated as pseudo
Substitution	2295801	2295801	1	3497393	3497393	1	G > A	-	*fsaB*	n/a	n/a	Irrelevant mutation, presence of an upstream frameshift in CDS
Substitution	2334436	2334436	1	3536028	3536028	1	C > A	-	Intergenic	-	-	-
Deletion	2334564	2334564	0	3536156	3549686	13,531	n/a	*rhaT; rhaR; rhaS; rhaB; rhaA; rhaD; yiiL; frvA; frvB; frvX; frvR; yiiG*	-	-	-	-
Deletion	2341355	2341355	0	3556478	3576238	19,761	n/a	*yiiF; yiiE; yiiD; dtd; rbn; yihX; yihW; yihV; yihU; yihT; yihS; yihR; yihQ; yihP; yihO; ompL; yihN; yihM; yihL*	-	-	-	-
Substitution	2433549	2433549	1	3668433	3668433	1	C > T	-	*rfe*	Missense	p.Met111Ile	-
Substitution	2449629	2449629	1	3684513	3684513	1	G > A	-	*ilvG*	n/a	n/a	Irrelevant mutation, presence of an upstream frameshift in CDS; restoration of *ilvG* found in MG1655
Substitution	2449740	2449740	1	3684624	3684624	1	C > A	-	*ilvG*	n/a	n/a	Irrelevant mutation, presence of an upstream frameshift in CDS; restoration of *ilvG* found in MG1655
Substitution	2449759	2449759	1	3684643	3684643	1	T > G	-	*ilvG*	n/a	n/a	Irrelevant mutation, presence of an upstream frameshift in CDS; restoration of *ilvG* found in MG1655
Substitution	2449803	2449803	1	3684687	3684687	1	A > G	-	*ilvG*	n/a	n/a	Irrelevant mutation, presence of an upstream frameshift in CDS; restoration of *ilvG* found in MG1655
Substitution	2449821	2449821	1	3684705	3684705	1	G > A	-	*ilvG*	n/a	n/a	Irrelevant mutation, presence of an upstream frameshift in CDS; restoration of *ilvG* found in MG1655
Substitution	2449833	2449833	1	3684717	3684717	1	A > G	-	*ilvG*	n/a	n/a	Irrelevant mutation, presence of an upstream frameshift in CDS; restoration of *ilvG* found in MG1655
Substitution	2449974	2449974	1	3684858	3684858	1	A > G	-	*ilvG*	n/a	n/a	Irrelevant mutation, presence of an upstream frameshift in CDS; restoration of *ilvG* found in MG1655
Substitution	2449986	2449986	1	3684870	3684870	1	C > T	-	*ilvG*	n/a	n/a	Irrelevant mutation, presence of an upstream frameshift in CDS; restoration of *ilvG* found in MG1655
Substitution	2450001	2450001	1	3684885	3684885	1	T > C	-	*ilvG*	n/a	n/a	Irrelevant mutation, presence of an upstream frameshift in CDS; restoration of *ilvG* found in MG1655
Substitution	2450037	2450037	1	3684921	3684921	1	A > G	-	*ilvG*	n/a	n/a	Irrelevant mutation, presence of an upstream frameshift in CDS; restoration of *ilvG* found in MG1655
Substitution	2450043	2450043	1	3684927	3684927	1	A > G	-	*ilvG*	n/a	n/a	Irrelevant mutation, presence of an upstream frameshift in CDS; restoration of *ilvG* found in MG1655
Substitution	2450154	2450154	1	3685038	3685038	1	C > T	-	*ilvG*	n/a	n/a	Irrelevant mutation, presence of an upstream frameshift in CDS; restoration of *ilvG* found in MG1655
Substitution	2450216	2450216	1	3685100	3685100	1	A > C	-	*ilvG*	n/a	n/a	Irrelevant mutation, presence of an upstream frameshift in CDS; restoration of *ilvG* found in MG1655
Substitution	2450245	2450245	1	3685129	3685129	1	T > A	-	*ilvG*	n/a	n/a	Irrelevant mutation, presence of an upstream frameshift in CDS; restoration of *ilvG* found in MG1655
Insertion	2450262	2450263	2	3685145	3685145	0	n/a	-	*ilvG*	Frameshift	p.Glu327_Ilefs*223	Restoration of complete *ilvG* found in MG1655 (548 amino acids), fusion of N-ter and C-ter fragments
Substitution	2450385	2450385	1	3685267	3685267	1	C > T	-	*ilvG*	Silent	p.Ala285Ala	-
Substitution	2450430	2450430	1	3685312	3685312	1	G > A	-	*ilvG*	Silent	p.Gly270Gly	-
Substitution	2450447	2450447	1	3685329	3685329	1	G > A	-	*ilvG*	Silent	p.Leu265Leu	-
Substitution	2450451	2450451	1	3685333	3685333	1	A > G	-	*ilvG*	Silent	p.Cys263Cys	-
Substitution	2450472	2450472	1	3685354	3685354	1	T > C	-	*ilvG*	Silent	p.Ala256Ala	-
Substitution	2450487	2450487	1	3685369	3685369	1	G > A	-	*ilvG*	Silent	p.His251His	-
Substitution	2450493	2450493	1	3685375	3685375	1	C > T	-	*ilvG*	Silent	p.Gly249Gly	-
Deletion	2462424	2462424	0	3697305	3709526	12,222	n/a	*rbsR; rbsK; rbsB; rbsC; rbsA; rbsD; trkD; yieN; yieM; asnA*	*hsrA*	Deletion	p.Val413_Glu475	*hsrA* is now annotated as pseudo
Deletion	2483011	2483011	0	3730114	3742029	11,916	n/a	*bglG; bglF; bglB; bglH; yieL; yieK; cbrC; yieI; yieH; yieG; yieF*	-	-	-	-
Deletion	2483916	2483916	0	3742936	3744271	1,336	n/a	*insD; insC*	-	-	-	-
Deletion	2486073	2486073	0	3746428	3751980	5,553	n/a	*'tnaB; insH; tnaB'; tnaA; insH; tnaC*	-	-	-	-
Deletion	2497001	2497001	0	3762909	3772816	9,908	n/a	*yidB; yidA; yidX; dgoR; dgoK; dgoA; dgoD; dgoT; cbrA; yidR; yidQ*	-	-	-	-
Substitution	2497023	2497023	1	3772839	3772839	1	C > A	-	Intergenic	-	-	-
Deletion	2498272	2498272	0	3774088	3785257	11,170	n/a	*yidE; yidP; glvC; glvB; glvG; ysdC; yidL; yidK; yidJ; yidI; yidH; yidG*	*yidF*	Deletion	p.Met1_Lys152del	*yidF* is now annotated as pseudo
Deletion	2507702	2507702	0	3794688	3803458	8,771	n/a	*ade; yicO; yicN; yicM; yicS; nlpA; yicL; setC*	-	-	-	-
Substitution	2528521	2528521	1	3824278	3824278	1	T > C	-	*rph*	Missense	p.Val138Ala	-
Substitution	2528567	2528567	1	3824324	3824324	1	T > C	-	*rph*	Silent	p.Asn153Asn	-
Substitution	2528603	2528603	1	3824360	3824360	1	A > T	-	*rph*	Silent	p.Gly165Gly	-
Substitution	2528609	2528609	1	3824366	3824366	1	G > A	-	*rph*	Silent	p.Val167Val	-
Substitution	2528615	2528615	1	3824372	3824372	1	C > T	-	*rph*	Silent	p.Gly169Gly	-
Substitution	2528621	2528622	2	3824378	3824379	2	GG > AA	-	*rph*	Silent; missense	p.Ala171Ala; p.Val172Ile	-
Substitution	2528651	2528651	1	3824408	3824408	1	T > A	-	*rph*	Silent	p.Ser181Ser	-
Substitution	2528755	2528755	1	3824512	3824512	1	T > C	-	*rph*	Missense	p.Ile216Thr	-
Substitution	2528765	2528765	1	3824522	3824522	1	T > G	-	*rph*	Silent	p.Ala219Ala	-
Insertion	2528779	2528779	1	3824535	3824535	0	n/a	-	*rph*	Frameshift	p.Glu224_Glyfs*16	*rph* is now annotated as pseudo
Substitution	2529025	2529025	1	3824781	3824781	1	T > C	-	*pyrE*	Silent	p.Asp45Asp	Restoration of *pyrE* found in MG1655
Substitution	2529058	2529058	1	3824814	3824814	1	G > A	-	*pyrE*	Silent	p.Ala56Ala	Restoration of *pyrE* found in MG1655
Substitution	2529070	2529070	1	3824826	3824826	1	C > T	-	*pyrE*	Silent	p.Ser60Ser	Restoration of *pyrE* found in MG1655
Substitution	2529124	2529124	1	3824880	3824880	1	C > G	-	*pyrE*	Silent	p.Ala78Ala	Restoration of *pyrE* found in MG1655
Substitution	2529130	2529130	1	3824886	3824886	1	A > T	-	*pyrE*	Silent	p.Thr80Thr	Restoration of *pyrE* found in MG1655
Substitution	2529136	2529136	1	3824892	3824892	1	T > C	-	*pyrE*	Silent	p.Ala82Ala	Restoration of *pyrE* found in MG1655
Substitution	2529142	2529142	1	3824898	3824898	1	A > G	-	*pyrE*	Silent	p.Ala84Ala	Restoration of *pyrE* found in MG1655
Substitution	2529157	2529157	1	3824913	3824913	1	C > T	-	*pyrE*	Silent	p.His89His	Restoration of *pyrE* found in MG1655
Substitution	2529163	2529163	1	3824919	3824919	1	G > T	-	*pyrE*	Silent	p.Leu91Leu	Restoration of *pyrE* found in MG1655
Substitution	2529193	2529193	1	3824949	3824949	1	A > G	-	*pyrE*	Silent	p.Glu101Glu	Restoration of *pyrE* found in MG1655
Substitution	2529295	2529295	1	3825051	3825051	1	G > A	-	*pyrE*	Silent	p.Glu135Glu	Restoration of *pyrE* found in MG1655
Substitution	2529316	2529316	1	3825072	3825072	1	C > T	-	*pyrE*	Silent	p.Ala142Ala	Restoration of *pyrE* found in MG1655
Substitution	2529340	2529340	1	3825096	3825096	1	G > A	-	*pyrE*	Silent	p.Val150Val	Restoration of *pyrE* found in MG1655
Substitution	2529352	2529352	1	3825108	3825108	1	C > T	-	*pyrE*	Silent	p.Leu154Leu	Restoration of *pyrE* found in MG1655
Substitution	2529367	2529367	1	3825123	3825123	1	C > G	-	*pyrE*	Silent	p.Arg159Arg	Restoration of *pyrE* found in MG1655
Substitution	2529448	2529448	1	3825204	3825204	1	C > T	-	*pyrE*	Silent	p.Asp186Asp	Restoration of *pyrE* found in MG1655
Substitution	2529457	2529457	1	3825213	3825213	1	T > C	-	*pyrE*	Silent	p.Ala189Ala	Restoration of *pyrE* found in MG1655
Substitution	2529460	2529460	1	3825216	3825216	1	C > T	-	*pyrE*	Silent	p.Tyr190Tyr	Restoration of *pyrE* found in MG1655
Substitution	2575671	2575671	1	3871427	3871427	1	G > A	-	Intergenic	-	-	-
Deletion	2576021	2576021	0	3871777	3914528	42,752	n/a	*yibG; yibJ; yibA; rhsA; yibF; selA; selB; yiaY; aldB; yiaW; yiaV; yiaU; yiaT; sgbE; sgbU; sgbH; lyxK; yiaO; yiaN; yiaM; yiaL; yiaK; yiaJ; yiaI; avtA; malS; bax; xylR; xylH; xylG; xylF; xylA; xylB; yiaB; yiaA; yiaH*	-	-	-	-
Substitution	2576125	2576125	1	3914633	3914633	1	A > G	-	Intergenic	-	-	-
Substitution	2576173	2576173	1	3914681	3914681	1	C > T	-	Intergenic	-	-	-
Deletion	2579583	2579583	0	3918091	3969123	51,033	n/a	*insK; insJ; hokA; cspA; yiaG; yiaF; tiaE; yiaD; bisC; yiaC; tag; yhjY; yhjX; eptB; proK; dppA; dppB; dppC; dppD; dppF; yhjV; rdlD; ldrD; bcsG; bcsF; bcsE; yhjR; yhjQ; bcsA; bcsB; bcsZ; bcsC; yhjK; dctA; yhjJ; kdgK; yhjH; yhjG; yhjE; yhjD; yhjC; yhjB*	-	-	-	-
Deletion	2597527	2597527	0	3987068	3989225	2,158	n/a	*insH; yhiS*	-	-	-	-
Substitution	2611280	2611280	1	4002979	4002979	1	C > T	-	Intergenic	-	-	-
Deletion	2611289	2611289	0	4002989	4002989	1	n/a	Intergenic	Intergenic	-	-	-
Deletion	2611305	2611305	0	4003005	4021346	18,342	n/a	*yhiN; yhiM; yhiL; yhiK; yhiJ; yhiI; rbbA; yhhJ; yhhI; yrhC; yhhH; rhsB*	-	-	-	-
Deletion	2645330	2645330	0	4055372	4061465	6,094	n/a	*yrhB; insB; insA; yrhA; yhhZ; yhhY; ryhB; yhhX; yhhW*	-	-	-	-
Substitution	2712097	2712097	1	4128233	4128233	1	A > G	-	*yhfZ*	Silent	p.Glu54Glu	-
Deletion	2712155	2712155	0	4128291	4141015	12,725	n/a	*yhfY; yhfX; yhfW; php; yhfU; yhfT; yhfS; frlR; frlD; frlC; frlB; frlA; yhfL*	*yhfZ*	Deletion	p.Met74_Tyr301del	*yhfZ* is now annotated as pseudo
Substitution	2712162	2712162	1	4141023	4141023	1	A > C	-	Intergenic	-	-	-
Substitution	2712164	2712164	1	4141025	4141025	1	C > A	-	Intergenic	-	-	-
Deletion	2741702	2741702	0	4170563	4186908	16,346	n/a	*chiA; bfd; bfr; gspO; gspM; gspL; gspK; gspJ; gspI; gspH; gspG; gspF; gspE; gspD; gspC; gspA; pioO*	-	-	-	-
Substitution	2794033	2794033	1	4239241	4239241	1	T > C	-	Intergenic	-	-	-
Insertion	2794287	2794338	52	4239494	4239494	0	n/a	-	Intergenic	-	-	-
Deletion	2794339	2794339	0	4239495	4255870	16,376	n/a	*yjbE; yjbF; yjbG; yjbH; yjbA; xylE; malG; malF; malE; malK; lamB; malM; yjbI*	-	-	-	-
Deletion	2819944	2819944	0	4281477	4281477	1	n/a	-	*soxR*	Frameshift	p.Gly140_Glufs*8	*soxR* is now annotated as pseudo
Deletion	2819965	2819965	0	4281498	4291254	9,757	n/a	*ryjA; yjcD; yjcE; yjcF; actP; yjcH; acs*	*soxR*	Deletion	n/a	Irrelevant mutation, presence of an upstream frameshift in CDS
Deletion	2826782	2826782	0	4298072	4366613	68,542	n/a	*gltP; yjcO; fdhF; yjcP; yjcQ; yjcR; yjcS; insH; alsK; alsE; alsC; alsA; alsB; rpiR; rpiB; yjdP; phnP; phnO; phnN; phnM; phnL; phnK; phnJ; phnI; phnH; phnG; phnF; phnE; phnD; phnC; phnB; phnA; yjdA; yjcZ; proP; basS; basR; eptA; adiC; adiY; adiA; melR; melA; melB; yjdF; fumB; dcuB; dcuR; dcuS; yjdI; yjdJ; yjdK; yjdO; lysU; yjdL; cadA; cadB; cadC*	-	-	-	-
Substitution	2826812	2826812	1	4366644	4366644	1	A > G	-	Intergenic	-	-	-
Substitution	2827055	2827055	1	4366887	4366887	1	G > A	-	Intergenic	-	-	-
Deletion	2831441	2831441	0	4371274	4371275	2	n/a	Intergenic	Intergenic	-	-	-
Substitution	2874927	2874927	1	4414761	4414761	1	T > A	-	Intergenic	-	-	-
Deletion	2874934	2874934	0	4414768	4428387	13,620	n/a	*yjfI; yjfJ; yjfK; yjfL; yjfM; yjfC; aidB; yjfN; yjfO; yjfP; ulaR; ulaG; ulaA; ulaB; ulaC; ulaD; ulaE*	*ulaF*	Deletion	p.Met1_Lys3del	*ulaF* is now annotated as pseudo
Deletion	2893608	2893608	0	4447062	4453719	6,658	n/a	*ytfM; ytfN; ytfP; yzfA; chpS; chpB*	-	-	-	-
Substitution	2894598	2894598	1	4454710	4454710	1	A > C	-	*ytfQ*	Silent	p.Pro23Pro	-
Deletion	2941326	2941326	0	4501438	4514770	13,333	n/a	*intB; insC; insD; yjgW; yjgX; yjgZ; insG; yjhB; yjhC; yjhD; yjhE; insN; insI; insM; insO; yjhW*	*yjhV*	Deletion	p.Met1_Glu124del	*yjhV* is now annotated as pseudo
Substitution	2949591	2949591	1	4523036	4523036	1	A > G	-	Intergenic	-	-	-
Deletion	2949669	2949669	0	4523114	4604874	81,761	n/a	*insA; insB; yjhU; yjhF; yjhG; yjhH; yjhI; sgcR; sgcE; sgcA; sgcQ; sgcC; sgcB; sgcX; yjhP; yjhQ; yjhX; yjhR; yjhS; yjhT; yjhA; fimB; fimE; fimA; fimI; fimC; fimD; fimF; fimG; fimH; gntP; uxuA; uxuB; uxuR; yjiC; yjiD; yjiE; iadA; yjiG; yjiH; kptA; yjiJ; yjiK; yjiL; yjiM; yjiN; yjiO; yjiP; yjiQ; yjiR; yjiS; yjiT; yjiV; mcrC; mcrB; yjiW; hsdS; hsdM; hsdR; mrr; yjiA; yjiX; yjiY; tsr; yjiZ; yjjM; yjjN; mdoB; yjjA*	-	-	-	-

^*a*^ Targeted deletions previously performed to generate DGF-298 were annotated as “Deletion_scar” in DGF-298 GenBank annotation file (CP127119.1).

^*b*^ GenBank CP127119.1; 08-JUN-2023.

^*c*^ GenBank AP009048.1; 07-OCT-2016.

^*d*^ Substitution only; n/a, non-applicable.

^*e*^ Genes were considered completely deleted below 10 remaining amino acids.

^*f*^ Pseudo qualifier (′) was added to GenBank features with the following mutation types: frameshift; mutation in translation start or stop codon; partial gene deletion; new upstream stop codon.

The final DGF-298 genome consists of a single circular chromosome of 2,991,126 bp and 2,831 genes, with an overall genome coverage of ~100× and a G + C content of ~51%.

## Data Availability

This Whole-Genome Shotgun project has been deposited in GenBank under the accession no. CP127119. The version described in this paper is the first version, CP127119.1. Raw ONT and Illumina reads have been deposited in Sequence Read Archive under the accession nos. SRR24843436 and SRR24843435, respectively.
